# Digital risk perception among middle-aged and older adults in China: a perspective from mobile phone dependence within the I-PACE model

**DOI:** 10.3389/fpsyt.2026.1746285

**Published:** 2026-02-06

**Authors:** Ying Lu, Rou Zhang, Weihui Dai

**Affiliations:** 1 College of Resources and Environment Engineering, Wuhan University of Science and Technology, Wuhan, China; 2 School of Management, Fudan University, Shanghai, China

**Keywords:** digital risk perception, fear of missing out, I-PACE model, mobile phone dependence, shap

## Abstract

**Introduction:**

The digital inclusion of middle-aged and older adults, while socially beneficial, exposes this population with relatively lower digital literacy to increased cybersecurity risks. However, the current understanding of the psychosocial mechanisms that undermine their digital risk perception (DRP) remains fragmented.

**Methods:**

Grounded in the Interaction of Person-Affect-Cognition-Execution (I-PACE) model, this study explored the interrelations among key psychological and behavioral factors associated with DRP. Data from 356 Chinese middle-aged and older adults aged 45 years and above in Chongqing were analyzed using structural equation modeling (SEM) combined with regression-based SHAP analysis.

**Results:**

Mobile phone dependence (MPD) was negatively associated with DRP (b = -0.253, p < 0.001). Loneliness and low perceived social support were correlated with greater fear of missing out (FoMO) and higher MPD, which were in turn linked to lower DRP. The findings also revealed a correlational pattern suggesting bidirectional tendencies between MPD and FoMO. The SHAP interpretation indicated that loneliness was the strongest correlate of DRP, followed by MPD and FoMO.

**Discussion:**

The identified pattern aligns with the process logic of the I-PACE framework. Overall, the study delineates an integrated pattern of psychological and behavioral factors influencing DRP and provides a conceptual basis for strengthening digital safety awareness and literacy among middle-aged and older adults.

## 1 Introduction

The aging of China’s population is accelerating at a notable pace (1). By 2020, adults aged 60 and older constituted 18.70% of the population, a cohort known as the “silver generation” (2), with projections indicating this will surpass 20% by 2032.This demographic shift is not merely a statistical trend but a profound social transformation, mirroring global patterns of population aging while presenting unique challenges and opportunities within the Chinese context (3). Concurrently, the digital revolution has swept across the nation, fundamentally altering how individuals live, work, and socialize. middle-aged and older adults have become the “second wave of digital mainstream users” (4). As of June 2024, individuals aged 40 and above accounted for half of all Chinese internet users (5), engaging routinely in online activities like chatting, shopping, and investing. This rapid digital integration reflects a broader global phenomenon where older adults are increasingly adopting digital technologies to maintain social connections, access services, and enhance their quality of life (6).

However, this digital inclusion is coupled with heightened vulnerability. Due to relatively limited digital skills, this demographic is particularly susceptible to online fraud (7), digital threats (8), and disruptive misinformation (9). Their first line of defense against these threats is digital risk perception (DRP)-that is, their ability to perceive, assess, and respond to online security threats, which enables the translation of safety awareness into actual protective behaviors (10). Yet, this critical cognitive ability is not formed in a vacuum and can be significantly eroded by maladaptive patterns of mobile device use.

Indeed, mobile phones have become the central medium for internet access, leading to prominent mobile phone dependence (MPD) among middle-aged and older adults-a condition characterized by a compulsive and uncontrolled urge to use the device, leading to negative impacts on daily life (11). The development of MPD is not random; it often follows a discernible psychological pathway rooted in fundamental human needs. This dependence often follows a clear psychological pathway: Loneliness (LON)-the subjective distress from perceived deficiencies in social relationships (12) -may propel individuals to seek connection through their phones. This need is intensified by fear of missing out (FoMO)-a pervasive anxiety about missing out on rewarding experiences others are having (13)-which fuels compulsive checking. Recent neuroimaging studies have shown that FoMO activates brain regions associated with social pain and reward anticipation, creating a powerful drive for continuous digital engagement, (14). Once entrenched, a cognitive pattern like ruminative thinking (RUM)-a repetitive, passive focus on one’s negative emotions and problems (15)-can deepen negative affect, reinforcing the dependence as a maladaptive coping mechanism. In contrast to these risk factors, the role of the protective factor, perceived social support (PSS)-an individual’s subjective evaluation of the availability and quality of support from their social network (16)-remains underexplored. However, longitudinal research indicates that higher levels of perceived social support are associated with lower subsequent psychological distress, suggesting its potential to mitigate the negative emotional states that often underlie compulsive digital behaviors (17).

Existing research on digital risk perception has primarily focused on younger populations, such as adolescents, whose overconfidence may lower their perceived risk (18), and has established that affective elements like FoMO can diminish threat sensitivity (19). Similarly, studies on mobile phone dependence predominantly concentrate on younger demographics and isolated psychological variables like anxiety (20).and loneliness (21); Consequently, significant gaps remain: (a) a lack of systematic investigation into the pathways shaping DRP in middle-aged and older adults; (b) an absence of an integrative theoretical framework explaining the relationship between mobile phone dependence and digital risk perception in this demographic; and (c) a failure to establish a coherent pathway model connecting personal traits, affective states, cognitive appraisal, and behavioral outcomes. Moreover, the unique socio-cultural context of China’s rapidly digitalizing society, combined with its distinct familial and social support structures for older adults, necessitates tailored investigations that existing Western-centric models may not fully capture (22).

To address these gaps, this study focuses on middle-aged and older adults, employing the I-PACE (Interaction of Person-Affect-Cognition-Execution) model as a theoretical framework to examine how individual traits (loneliness and perceived social support), emotional needs (fear of missing out), and cognitive factors (ruminative response) are associated with digital risk perception through the potential mediating role of mobile phone dependence. By doing so, it aims to explore the psychological pathways associated with digital risk perception, providing a conceptual basis to better understand and enhance middle-aged and older adults’ capacity to cope with online threats. This research not only addresses a critical gap in the literature but also responds to an urgent societal need to support a digitally disadvantaged and expanding population in the digital age.

## 2 The I-PACE theoretical framework

### 2.1 Overview of the model

**Figure 1:**
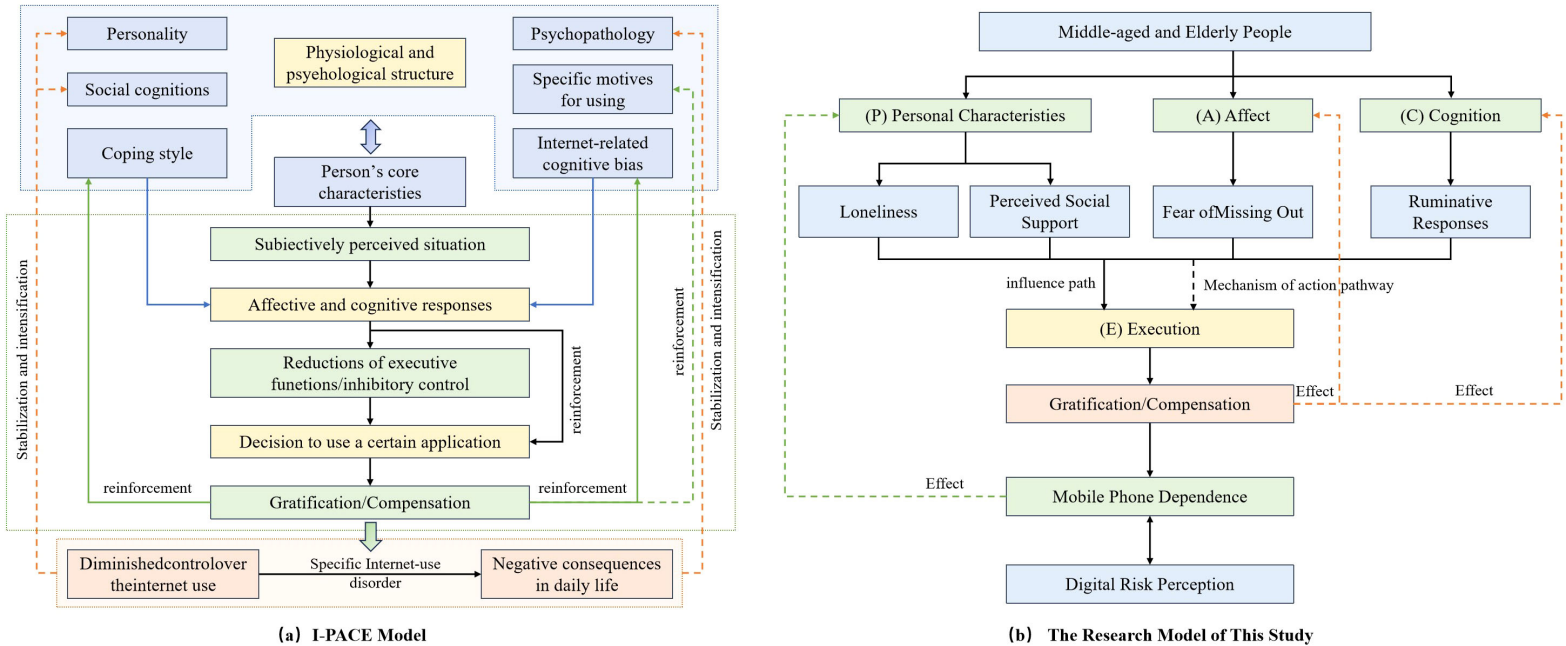
Theoretical research model.

The Interaction of Person-Affect-Cognition-Execution (I-PACE) model (**Figure 1a**), developed by Brand et al. (23), provides a comprehensive framework for understanding the development and maintenance of specific internet-use disorders and other behavioral addictions. The model posits that such maladaptive behaviors are the outcome of a dynamic, iterative process involving the interplay of four core components.

According to the model, relatively stable predisposing personal characteristics (P), such as personality traits, psychopathology, or specific vulnerabilities-form the foundation. These predispositions influence an individual’s affective (A) and cognitive (C) responses to internal and external cues. The affective component encompasses immediate emotional reactions, while the cognitive component involves processes like attention bias, expectancies, and decision-making. The interplay between affect and cognition subsequently impacts executive functions (E), which include inhibitory control, decision-making, and behavioral regulation as illustrated. A central tenet of the I-PACE model is that repeated engagement in the behavior can lead to a weakening of executive control and a simultaneous strengthening of cue-reactivity, thereby creating a self-reinforcing cycle that perpetuates the problematic behavior over time. Due to its systematic approach, the I-PACE model has been widely applied to research on internet addiction, gaming disorder, and compulsive social media use. (24).

### 2.2 Conceptual framework and hypotheses development

Based on the I-PACE theoretical framework, this study constructs a conceptual path model (**Figure 1b**) to delineate the mechanisms through which personality traits, affective responses, and cognitive patterns are associated with mobile phone dependence (MPD) and digital risk perception (DRP) among middle-aged and older adults.

The model situates loneliness (LON) and perceived social support (PSS) as core person-level variables, positing that they directly shape emotion-driven compensatory behaviors related to smartphone use.

At the affective level, fear of missing out (FoMO) is theorized to be linked with heightened and more frequent engagement with online content, thereby reinforcing compulsive digital routines.

On the cognitive plane, ruminative thinking (RUM) is hypothesized to relate to weaker executive inhibition, increasing the likelihood of impulsive online actions and reducing vigilance toward digital risks.

Overall, the path model suggests that the interaction between personal traits and emotional needs may contribute to the formation of MPD. In parallel, deficits in executive control-reinforced by such dependence-are expected to be associated with a reduced capacity for accurate digital risk perception, completing a pathway from internal dispositions to external behavioral outcomes.

Based on the aforementioned path analysis and theoretical framework, this study proposes the following research hypotheses:

H1: (a)LON, (b)PSS, and (c)RUM have significant effects on FoMO.
H2: (a)LON, (b)PSS, and (c)RUM have significant effects on MPD.
H3: LON, PSS, RUM, FoMO, and MPD collectively exert significant effects on DRP.
H4: FoMO mediates the relationships between (a) LON, (b) PSS, (c) RUM and DRP.
H5: MPD mediates the relationships between (a) LON, (b) PSS, (c) RUM and DRP.

## 3 Methodology

### 3.1 Measurement scales

This study utilized a self-developed questionnaire along with five well-established psychological assessment instruments (**Table 1**). The Digital Risk Perception (DRP) Scale was developed based on the Knowledge-Attitude-Practice (KAP) model (27). The initial item pool was conceptually evaluated and iteratively refined through a literature review to ensure that each item accurately reflected the cognitive, affective, and behavioral components of digital risk awareness among middle-aged and older adults.

**Table 1:** Measurement Instruments.

Construct	Dimensions	Source
Digital Risk Perception	Knowledge, Attitudes, Behaviors	/
Mobile Phone Dependence	Loss of control, Avoidance, Withdrawal, Inefficiency	(25)
Loneliness	/	(26)
Perceived Social Support	Family, Friends, Significant others	(16)
Fear of Missing Out	Fear of missing information, Fear of missing experiences	(13)
Rumination	Symptom-focused, Reflective pondering, Compulsive meditation	(15)

The DRP scale was divided into two parts: the first part included five questions that collected basic demographic information, while the second part included twelve core questions related to digital risk perception. The core questions of the DRP scale consisted of twelve items, designed to cover three dimensions: knowledge (understanding of online risks), attitudes (concern and perceived severity), and behaviors (self-reported protective practices). Sample items include: for the Knowledge dimension, “*I am aware that setting a lock-screen password is an important way to protect personal privacy*”; for the Attitude dimension, “*I believe that connecting to free public Wi-Fi without a password requires great caution*”; and for the Behavior dimension, “*I set different passwords for different important accounts (e.g., WeChat, bank cards)*.”

The initial 12-item version (four items per dimension, including reverse-worded items) was administered. Specifically, the reverse-worded items (items 8, 11, and 15) were included to identify inconsistencies in responses and to ensure the reliability of the scale. The original scoring of the DRP scale was retained, where a five-point Likert scale was used for each item, with higher scores reflect lower levels of digital risk awareness and weaker vigilance toward online risks. Based on subsequent psychometric validation (detailed in Section 4.2), one item from the Knowledge dimension was removed to optimize the scale. Therefore, all analyses in this study are based on the final 11-item DRP scale (Knowledge: 3 items, Attitudes: 4 items, Behaviors: 4 items). For all subsequent analyses, the composite score (the average of the 11 items) was used as the measure of DRP. Sample items for each dimension and the full scale are provided as **Supplementary Material**.

A detailed psychometric evaluation of the DRP scale, including item analysis, exploratory and confirmatory factor analyses of its three-factor structure, and the final reliability indices, is presented in Section.

### 3.2 Data collection and descriptive statistics

Participants in this study were middle-aged and older adults aged 45 years and above, recruited in Chongqing, China, through a combination of on-site and online questionnaire administration. Before participation, each participant was verbally or in writing informed about the study. Participation was entirely voluntary, and confidentiality and anonymity were guaranteed. After excluding 22 invalid cases due to incomplete or inconsistent responses, 356 valid responses were retained, yielding a response rate of 94.17%. The demographic characteristics of the sample are summarized in **Table 2**.

**Table 2:** Demographic characteristics of respondents.

Attribute	Category	Sample size
Gender	Male	140
	Female	216
Region	Urban	237
	Township	119
Age Group	Middle-aged adults	306
	Older adults	50
Self-rated Health	Good	272
	Average	69
	Poor	15
Education Level	Primary school or below	26
	Junior high school	108
	High school/Vocational school	141
	College or above	81

### 3.3 Reliability and validity assessment

Before assessing the reliability and validity of the study measures, a test for potential common method bias (CMB) was conducted, as all variables were obtained through self-report questionnaires. Harman’s single-factor test was performed by subjecting all measurement items to an unrotated exploratory factor analysis. The first unrotated factor accounted for 33.572% of the total variance, which is below the commonly accepted threshold of 40%. Therefore, CMB was not considered a serious concern in this study, and the data were deemed suitable for subsequent reliability, validity, and structural analyses.

Following the assessment of common method bias, the reliability and validity of the questionnaire data were examined using SPSS 27, with detailed results presented in **Table 3**. Reliability was evaluated using Cronbach’s α coefficient, and all scales demonstrated excellent internal consistency, with α values exceeding 0.95. Validity was assessed through the Kaiser-Meyer-Olkin (KMO) measure and Bartlett’s test of sphericity. The KMO value was 0.964 (>0.50), and Bartlett’s test yielded a significance level of 0.000 (<0.05), indicating robust construct validity, a stable internal structure, and high measurement precision. To further ensure the psychometric soundness of the self-developed DRP scale, item analysis, exploratory and confirmatory factor analyses, and structural validation were subsequently performed; these procedures are described in detail in Section 4.2.

**Table 3:** Assessment of internal consistency and validity.

Dimension	Cronbach α	KMO
LON	0.985	0.964
PSS	0.983	
RUM	0.983	
FoMO	0.951	
MPD	0.970	
DRP	0.964	

### 3.4 Regression analysis with SHAP visualization

Multiple linear regression analysis was first conducted using SPSS (version 27.0) to examine the psychological predictors of DRP. The independent variables included LON, PSS, FoMO, RUM, and MPD.

In addition to reporting conventional inferential statistics (standardized coefficients, R², and p-values), SHAP (SHapley Additive exPlanations) values were computed based on the fitted regression model using Python (version 3.12). The SHAP analysis was applied to visualize the relative contribution of each predictor to the model’s prediction of DRP, emphasizing the importance of each predictor and the direction of its influence on DRP.

Importantly, the SHAP analysis in this study was interpretive rather than predictive, serving to illustrate the association patterns identified in the regression model without modifying its parameters, offering complementary insights into the determinants of DRP.

## 4 Results

### 4.1 Sociodemographic variables and digital risk perception in middle-aged and older adults

Differences in MPD and DRP across demographic variables were observed among middle-aged and older adults, which may lead to distinct behavioral patterns among different subgroups. Independent-samples t-tests were conducted to examine the relationship between demographic variables and DRP. Gender, age, educational level, place of residence, and self-rated health status were included as indicators. All subgroup sample sizes exceeded 30, and the data were normally distributed, satisfying the preconditions for parametric tests.

In **Table 4**, a one-way ANOVA revealed significant differences in MPD across age groups (F (2, 353) = 7.838, p < 0.001, η² = 0.043). Although the age groups were uneven in size, the analysis indicated a small-to-moderate effect, suggesting that MPD tended to increase slightly with age. This pattern may be associated with the growing adaptation to and reliance on mobile devices for social interaction, information access, and daily life support among middle-aged and older adults. It is important to note that the sample sizes in each group were not equal, which may influence the interpretation of the results.

**Table 4:** Group differences in mobile phone dependence across age groups.

Age	Mean	SD	η²	F	P
45-59	3.039	0.958	0.043	7.838	<0.001
60-70	3.320	0.736			
≥70	3.470	0.926			

In **Table 5**, a statistically significant difference in DRP was observed based on place of residence (t = 4.483, p < 0.001). This indicates that place of residence, as a key sociodemographic variable, is associated with substantial differences digital risk awareness. Previous research suggests that the digital divide extends beyond internet access to encompass disparities in skills and knowledge, often placing rural and older adult populations in a structurally disadvantaged position in this regard (28),These findings are consistent with the results of the present study. Urban residents demonstrated a relatively higher level of DRP, which may stem from more frequent exposure to digital technologies and greater encounters with complex cyber risks in urban environments. In contrast, the lower mean score observed in the rural group may reflect limited digital access and inadequate cultivation of risk awareness, resulting in a weaker perception of online threats. Furthermore, in terms of standard deviation, the urban group recorded an SD of 0.950, while the rural group had an SD of 0.897. Both values are relatively high, indicating substantial variability in DRP among individuals within both urban and rural populations.

**Table 5:** Differences in digital risk perception based on place of residence.

Residence	Mean	SD	t	P
Urban	2.910	0.950	4.483	<0.001
Rural	2.471	0.897		

Higher DRP scores indicate lower digital risk perception.

### 4.2 Validation of the digital risk perception scale

The core questions of the DRP scale included twelve items under the Knowledge-Attitude-Practice (KAP) framework. During preliminary analyses, one knowledge-dimension item-”*I understand that excessive sharing of daily life details (such as travel routes or home address) on social platforms like WeChat may lead to potential risks*”-showed weak performance. Its corrected item-total correlation (r = 0.42) and factor loading (0.52) were notably lower than acceptable levels, indicating limited contribution to the construct. During the on-site questionnaire administration, several middle-aged and older participants regarded “excessive sharing” as normal social interaction rather than a risk behavior, suggesting semantic ambiguity. Considering both statistical and conceptual evidence, this item was removed, yielding an eleven-item DRP scale that retained a coherent three-dimensional structure encompassing knowledge, attitudes, and protective behaviors.

To evaluate the scale’s construct validity, the revised 11-item DRP scale was subsequently examined using both exploratory factor analysis (EFA) and confirmatory factor analysis (CFA). The EFA revealed a clear three-factor structure consistent with the KAP framework. Sampling adequacy was excellent (KMO = 0.921; Bartlett’s χ² = 2823.460, df = 55, p < 0.001). All items loaded strongly on their intended dimensions (0.75-0.83), and the three factors together explained 78.87% of the total variance. **Table 6** summarizes the EFA results, confirming a theoretically coherent and statistically stable three-factor structure.

**Table 6:** Summary of exploratory factor analysis (EFA) results for the DRP scale (N = 356).

Dimension	Number of items	Loading range	Eigenvalue	Variance explained (%)	Cronbach’s α
Knowledge	3	0.75 - 0.81	6.423	58.40	0.918
Attitudes	4	0.83 - 0.88	1.201	10.92	0.932
Behaviors	4	0.84 - 0.89	1.052	9.56	0.940
Total	11	–	–	78.87	0.964

Extraction method: Principal component analysis with varimax rotation.KMO = 0.921; Bartlett’s χ²(55) = 2823.460, p < 0.001.

The subsequent CFA further verified this measurement model. As shown in **Table 7**, the three-factor model demonstrated an excellent overall fit (χ²/df = 1.651, RMSEA = 0.032, GFI = 0.967, AGFI = 0.946, NFI = 0.976, TLI = 0.987, CFI = 0.990), meeting recommended thresholds for good fit (χ²/df < 3, RMSEA < 0.05, CFI > 0.95). All standardized factor loadings were significant (p <.001) and ranged from 0.782 to 0.892, with moderate inter-factor correlations (r = 0.67-0.88), indicating clear discriminant validity.

**Table 7:** Confirmatory factor analysis (CFA) model fit indices.

Fit indices	χ²/df	RMSEA	GFI	AGFI	NFI	TLI	CFI
Model Fit	1.651	0.032	0.967	0.946	0.976	0.987	0.990

χ²/df < 3, RMSEA < 0.05, and CFI > 0.95 indicate excellent model fit.

As summarized in **Table 8**, the construct reliability and convergent validity of the three latent dimensions were satisfactory. The composite reliability (CR) values ranged from 0.86 to 0.90, and the average variance extracted (AVE) values exceeded 0.63, supporting good convergent validity. Internal consistency was excellent, with Cronbach’s α of 0.918 (knowledge), 0.932 (attitude), 0.940 (behavior), and 0.964 (total scale). Although reliability coefficients were high, indicating potential item overlap, this likely reflects the focused and homogeneous nature of the construct rather than problematic redundancy.

**Table 8:** Construct reliability and convergent validity of the DRP scale.

Construct	CR	AVE	Cronbach’s α	Loading Range
Knowledge	0.86	0.63	0.918	0.78 - 0.81
Attitudes	0.88	0.67	0.932	0.83 - 0.88
Behaviors	0.90	0.68	0.940	0.84 - 0.89

All standardized loadings significant at p < 0.001; inter-factor correlations r = 0.67 - 0.88.

Taken together, the EFA and CFA results provide robust empirical evidence supporting the three-dimensional structure of the DRP scale, thereby confirming its theoretical coherence and psychometric soundness in assessing DRP among middle-aged and older adults.

### 4.3 Validation of the full measurement model and bivariate correlations

Before testing the structural model, a CFA was conducted to evaluate the adequacy of the overall measurement model comprising all latent variables (LON, PSS, FoMO, RUM, MPD, and DRP). The six-factor measurement model demonstrated satisfactory fit to the data (χ²/df = 1.389, RMSEA = 0.033, CFI = 0.959, and TLI = 0.958). All standardized factor loadings were significant (p <.001) and exceeded 0.70, indicating good convergent validity. The average variance extracted (AVE) for each construct ranged from 0.63 to 0.78, and composite reliability (CR) values exceeded 0.85. These results confirm both the convergent and discriminant validity of the measurement model, supporting its adequacy for subsequent SEM analyses.

Correlation analysis among the variables is presented in **Table 9**. All correlation coefficients aligned with theoretical expectations, with absolute values ranging between 0.197 and 0.400, indicating low-to-moderate relationships. For instance, LON showed a significant negative correlation with PSS (r = -0.330, p < 0.01), suggesting that middle-aged and older adults with higher levels of LON tended to perceive lower social support. This pattern implies that pronounced feelings of LON may form a negative cognitive filter, potentially leading individuals to underestimate or overlook available social support.

**Table 9:** Correlation analysis.

Category	LON	PSS	RUM	FoMO	MPD	DRP
LON	–					
PSS	-0.330	–				
RUM	0.335	-0.330	–			
FoMO	0.314	-0.294	0.265	–		
MPD	0.355	-0.302	0.197	0.294	–	
DRP	-0.366	0.319	-0.320	-0.342	-0.400	–

Higher DRP scores indicate lower digital risk perception.

In accordance with the Fornell-Larcker criterion, the square roots of the AVE for all constructs (ranging from 0.782 to 0.827) exceeded their correlations with other constructs. For example, the square root of the AVE for LON (0.782) was substantially higher than its correlation with RUM (0.335). This correlation pattern not only supports the discriminant validity of the theoretical construct network but also provides a solid foundation for the subsequent path analysis within the structural equation modeling framework.

### 4.4 Structural equation modeling analysis: direct effect pathways

This study employed structural equation modeling (SEM) for data analysis with a sample size of 356. According to SEM guidelines, at least 10 participants per free parameter are recommended (29). Considering the model’s complexity and the number of indicators used, the sample size is sufficient to support reliable estimation of each indicator, ensuring robust model estimates and dependable analysis results.

**Figure 2:**
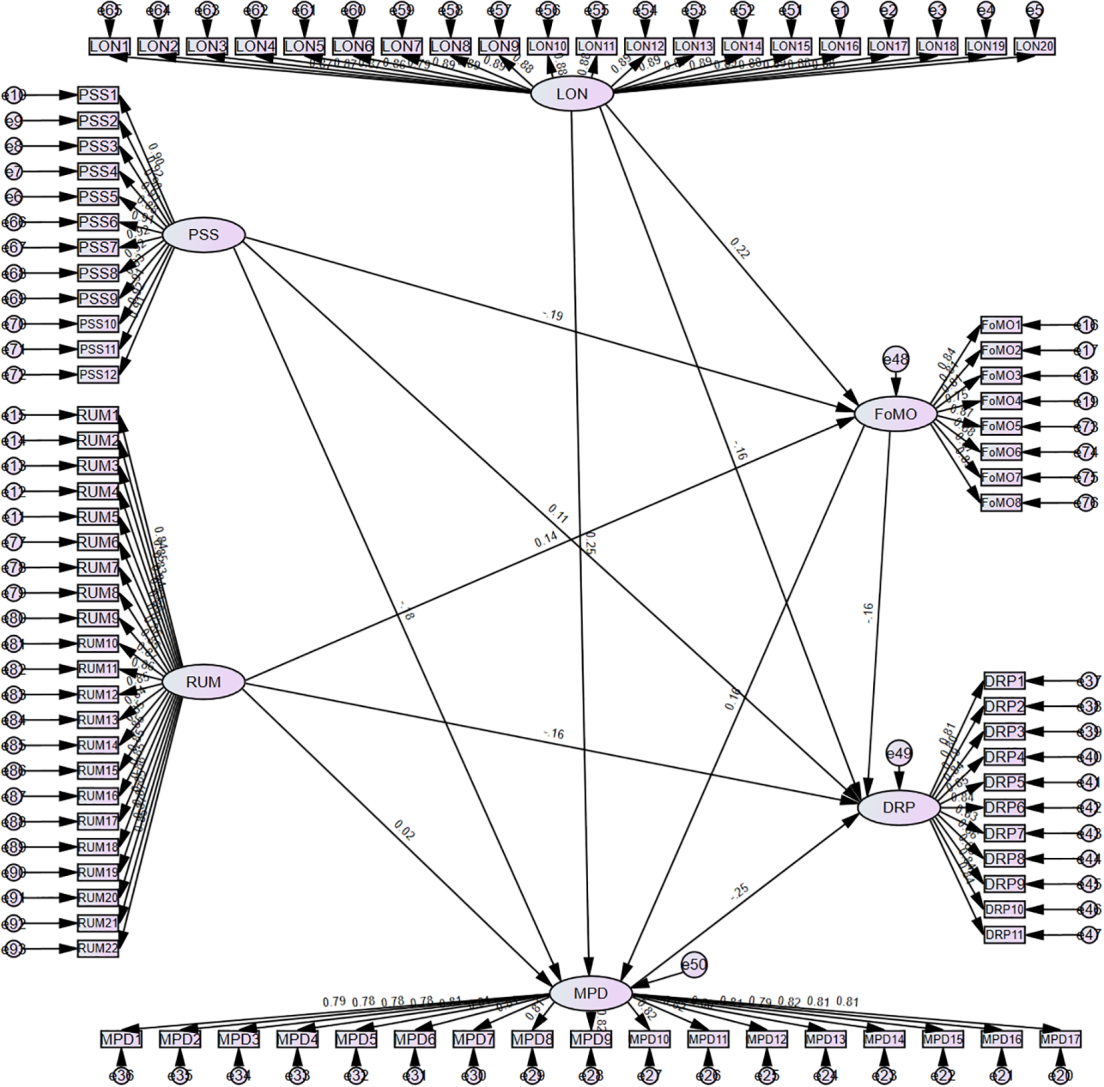
Structural equation model.

As illustrated in **Figure 2**, the structural equation modeling analysis demonstrated good model-data fit (χ²/df = 1.389, RMSEA = 0.033, TLI = 0.958, CFI = 0.959). The core individual traits of LON and PSS were confirmed as significant predictors of FoMO. Specifically, LON significantly exacerbated FoMO (β = 0.219, p < 0.001), while PSS effectively alleviated such anxiety (β = -0.192, p < 0.001), supporting Hypothesis H1. Furthermore, rumination demonstrated a significant positive effect on FoMO (β = 0.138, p = 0.009).The effect sizes were moderate, indicating that the observed relationships were meaningful but not excessively strong.

Regarding the predictors of MPD, the model yielded distinct patterns for the psychological traits. LON significantly reinforced MPD (β = 0.251, p < 0.001), while perceived social support served as a significant protective factor (β = -0.177, p < 0.001). In contrast, the direct path from ruminative thinking to MPD was not statistically significant (β = 0.019, p = 0.712). Consequently, Hypothesis H2, which proposed that (a) LON, (b) PSS, and (c) RUM all have significant effects on MPD, received partial support, being confirmed for LON and PSS but not for RUM.

DRP was found to be influenced by multidimensional factors in a complex manner. Apart from the modest protective effect of PSS (β = 0.107, p = 0.033),LON (β = -0.159, p = 0.002), RUM (β = -0.156, p = 0.002), FoMO (β = -0.163, p = 0.002), and MPD (β = -0.253, p < 0.001) all demonstrated significant negative effects. This network of pathways confirms the validity of Hypothesis H3.

### 4.5 SHAP analysis of regression results

The SEM results confirmed the hypothesized relationships among variables (**Figure 2**). To further clarify the direct contributions of specific predictors to DRP, a multiple regression analysis was conducted. The regression model was significant (F (5, 350) = 27.81, p < 0.001, R² = 0.284, Adjusted R² = 0.274), consistent with the SEM results, and the directions of the observed paths remained identical.

As shown in **Table 10**, LON (β = -0.149, p = 0.005), RUM (β = -0.148, p = 0.003), FoMO (β = -0.155, p = 0.002), and MPD (β = -0.242, p < 0.001) were significant negative predictors, indicating that higher levels of LON, RUM, FoMO, and MPD were associated with lower levels of DRP. In contrast, PSS (β = 0.102, p = 0.046) showed a small but significant positive association with DRP, suggesting that stronger perceived social support corresponds to greater awareness and vigilance toward online risks.

**Table 10:** Results of multiple regression analysis predicting DRP (N = 356).

Predictor	B	SE	β	t	p
Constant	4.639	0.315	–	14.736	<0.001
LON	-0.153	0.054	-0.149	-2.857	0.005
PSS	0.074	0.037	0.102	2.003	0.046
RUM	-0.145	0.049	-0.148	-2.963	0.003
FoMO	-0.160	0.051	-0.155	-3.114	0.002
MPD	-0.252	0.052	-0.242	-4.820	<0.001

(a) DRP, digital risk perception; LON, loneliness; PSS, perceived social support; RUM, rumination; FoMO, fear of missing out; MPD, mobile phone dependence. (b) Higher DRP scores indicate lower digital risk perception.

**Figure 3:**
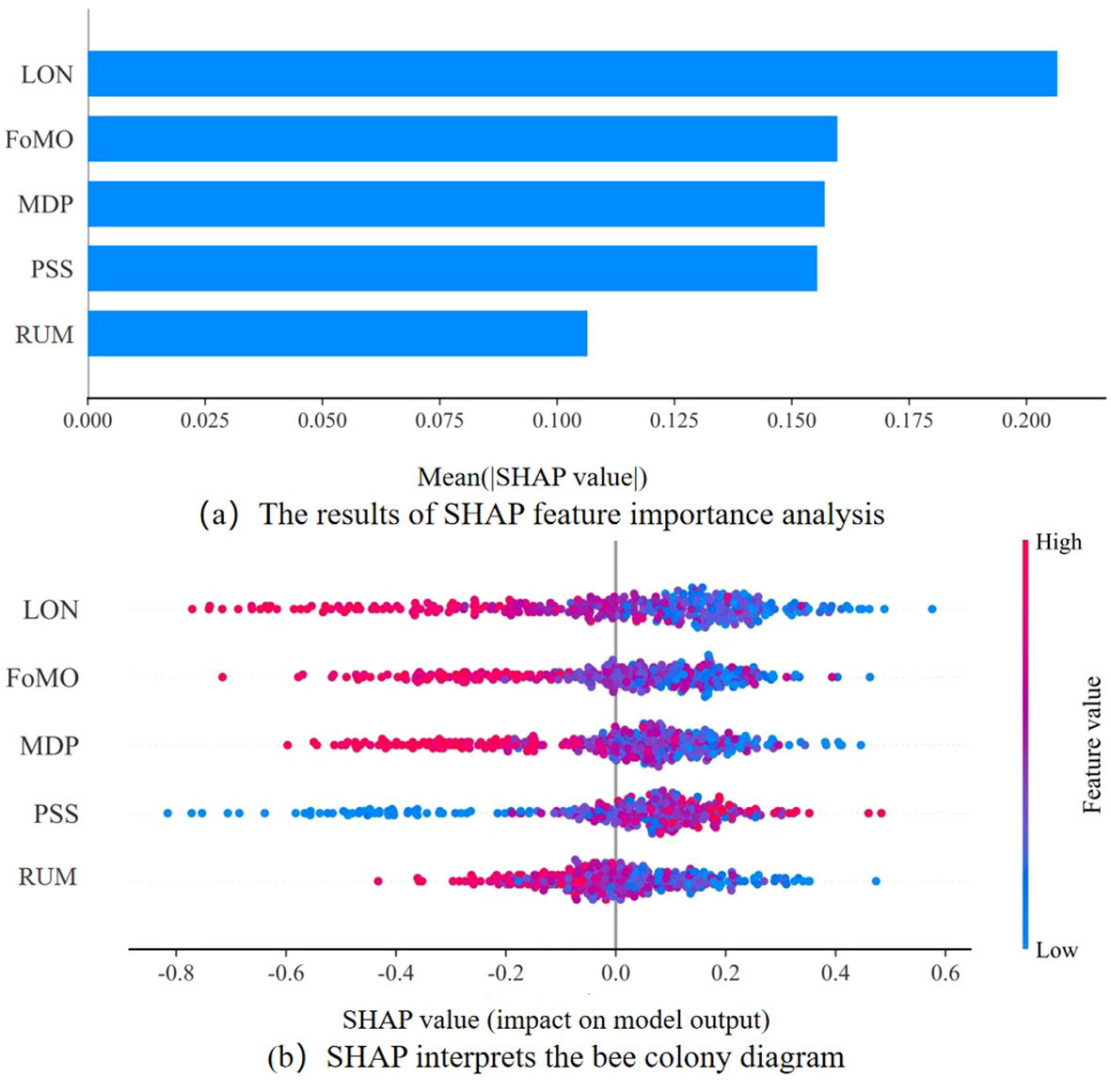
SHAP analysis of predictors of digital risk perception. Higher DRP scores indicate lower perceived digital risk.

To further examine and interpret these relationships, a SHAP analysis was conducted to visualize the regression model and estimate the relative contribution of each predictor. As shown in **Figure 3a**, LON was identified as the most influential predictor, displaying the highest mean SHAP value, followed by MPD and FoMO, while PSS and RUM demonstrated comparatively smaller contributions. **Figure 3b** illustrates the directional trends of these associations: LON, FoMO, and MPD were consistently linked to decreased risk perception (negative SHAP values), whereas PSS exhibited a protective pattern (positive SHAP values).

These SHAP based interpretations provide additional insights into the regression model, highlighting the relative importance of predictors such as LON, MPD, and FoMO in predicting DRP. Together with SEM, both analytical approaches complement each other in identifying key psychological factors associated with DRP among middle-aged and older adults, demonstrating that SHAP enhances the interpretability and clarity of regression outcomes.

### 4.6 Mediation analysis: indirect association pathways

The indirect pathways were examined using bootstrap sampling to assess the significance of mediating associations among variables. As summarized in **Table 11**, seven indirect paths showed statistically significant mediating effects (p < 0.05). Both FoMO and MPD were found to play significant mediating roles in the relationships between LON, PSS, and DRP. These findings highlight the multilayered psychological pathways through which individual characteristics are statistically associated with variations in DRP.

**Table 11:** Mediation effect analysis.

Path	Estimate	Lower	Upper	P
LON→FoMO→DRP	-0.036	-0.073	-0.012	0.004
PSS→FoMO→DRP	0.031	0.010	0.072	0.005
RUM→FoMO→DRP	-0.022	-0.051	-0.004	0.015
LON→MPD→DRP	-0.063	-0.118	-0.029	0.000
PSS→MPD→DRP	0.045	0.015	0.094	0.001
RUM→MPD→DRP	-0.005	-0.035	0.027	0.798
LON→FoMO→MPD→DRP	-0.009	-0.023	-0.003	0.002
PSS→FoMO→MPD→DRP	0.008	0.003	0.020	0.001
RUM→FoMO→MPD→DRP	-0.006	-0.017	-0.001	0.010

The mediation pathway through FoMO was statistically significant, supporting Hypothesis H4. LON, PSS, and RUM all showed significant indirect associations with DRP through their linkage with FoMO. The mediating role of MPD was also confirmed, as both LON and PSS demonstrated significant indirect relationships with DRP via this behavioral pathway. Consequently, Hypothesis H5 was partially supported, with the exception of the non-significant pathway from RUM to MPD to risk perception.

Furthermore, the analysis revealed an unexpected mediation pathway (FoMO → MPD). Both LON and PSS were found to be associated with lower DRP through FoMO and subsequently through MPD. This pattern illustrates a statistical sequence linking individual characteristics to emotional responses, behavioral dependence, and cognitive judgment. Rather than implying reciprocal or cyclic dynamics, the results highlight a structured sequence of associations shaping DRP among middle-aged and older adults. The results highlight a structured sequence of associations shaping DRP among middle-aged and older adults.

## 5 Discussion

### 5.1 A systemic framework: personal characteristics, affective response, and cognitive control

Regarding the measurement instrument, the self-developed DRP scale demonstrated very high internal consistency (α > 0.95) in the current sample, indicating excellent reliability for this exploratory study. However, such high alpha values may also suggest a degree of item redundancy, where multiple items are measuring extremely similar aspects of the construct (30). In our context, this could be attributable to the relatively focused and concrete nature of digital risk perception as operationalized for middle-aged and older adults. While this redundancy ensures measurement stability and is acceptable for the primary goal of this study-to establish a coherent psychological and behavioral pathway-it may limit the scale’s parsimony and efficiency for future applications. The particularly strong reliability and model fit indices observed for the DRP scale may partly reflect its conceptually focused structure and the relative homogeneity of the sample, rather than artificial inflation of measurement quality.

To enhance the validity and interpretability of the DRP scale, future research should further examine its psychometric properties. In particular, the construct validity of the scale can be strengthened by testing its convergent and discriminant validity, as well as by conducting confirmatory factor analyses to verify the dimensionality of the three proposed domains-knowledge, attitudes, and behaviors. Moreover, item-level refinements (e.g., rewording or removing overlapping items and introducing reverse-worded items) may help reduce redundancy and improve parsimony, ultimately enabling the DRP scale to serve as a more concise and robust instrument for assessing digital risk perception across diverse populations. Now that we have addressed the measurement considerations, we will focus on the theoretical interpretation of our findings, specifically examining how individual psychological characteristics influence digital risk perception among middle-aged and older adults.

(1) Personal characteristics (P): foundational drivers of the model: within the I-PACE framework, the model conceptualizes two pivotal yet contrasting person-level characteristics as foundational drivers of the process: LON acts as a risk-enhancing factor, while perceived social support functions as a protective buffer. This configuration underscores that the DRP of middle-aged and older adults is closely linked to the fundamental human need for social connection. LON, representing a deficit in social fulfillment, predisposes individuals to compensatory digital engagement by activating heightened affective and cognitive needs. Conversely, PSS serves as a stable psychological resource, mitigating the emotional strain associated with social disconnection. This dynamic is supported by empirical evidence (31), which demonstrated that perceived isolation can exacerbate mental health vulnerabilities. Consistent with this evidence, the present findings suggest that LON may diminish an individual’s perception of available social resources, thereby encouraging maladaptive coping behaviors such as excessive smartphone use.

(2) Affective responses (A): the emotional expression of psychological needs: the analysis confirms that personality traits are reflected at the affective level, with FoMO acting as a critical intervening factor. The significant P→A path indicates that LON is positively associated with higher levels of FoMO, whereas perceived social support shows a negative association with it. FoMO can be understood as a pervasive apprehension about missing rewarding experiences, which drives individuals toward compulsive engagement with digital platforms as a coping strategy for negative affectivity (32). Importantly, the identification of a significant direct A→E path (FoMO → DRP) indicates that this anxiety is linked to lower levels of DRP. This association is supported by a recent meta-analysis, which established a robust relationship between FoMO and a spectrum of negative outcomes, including problematic smartphone and social media use, as well as difficulties in emotional regulation (33). Within the context of our model, these findings suggest that the persistent need to maintain social connectedness may undermine executive control, with individuals potentially neglecting risk evaluation in favor of immediate emotional gratification.

(3) Cognitive structure and control (C): the cognitive basis of behavioral dysregulation: The model further illustrates how affective responses are reflected in maladaptive cognitive tendencies, with MPD serving as the central construct. The significant A→C path indicates that FoMO is positively associated with higher levels of MPD, as individuals may increasingly turn to their devices to alleviate anxiety. The C→E path shows that MPD, as a core cognitive-behavioral correlate, is linked to lower levels of DRP. This pattern is supported by recent empirical evidence. A systematic review by (34) reported that excessive smartphone use is associated with various health and cognitive difficulties, including reduced self-control and attentional lapses-factors that may help explain the diminished vigilance toward online risks observed in this study. The global relevance of this issue is highlighted by a recent meta-analysis (35), which identified smartphone overuse as a widespread and growing concern across diverse populations and cultural contexts. An important observation from our model concerns the potential C→A reciprocal pathway, wherein established MPD appears to be related to heightened FoMO. This mutual reinforcement may represent a self-sustaining behavioral pattern, gradually reducing individuals’ attentional resources for effective digital risk assessment.

**Figure 4:**
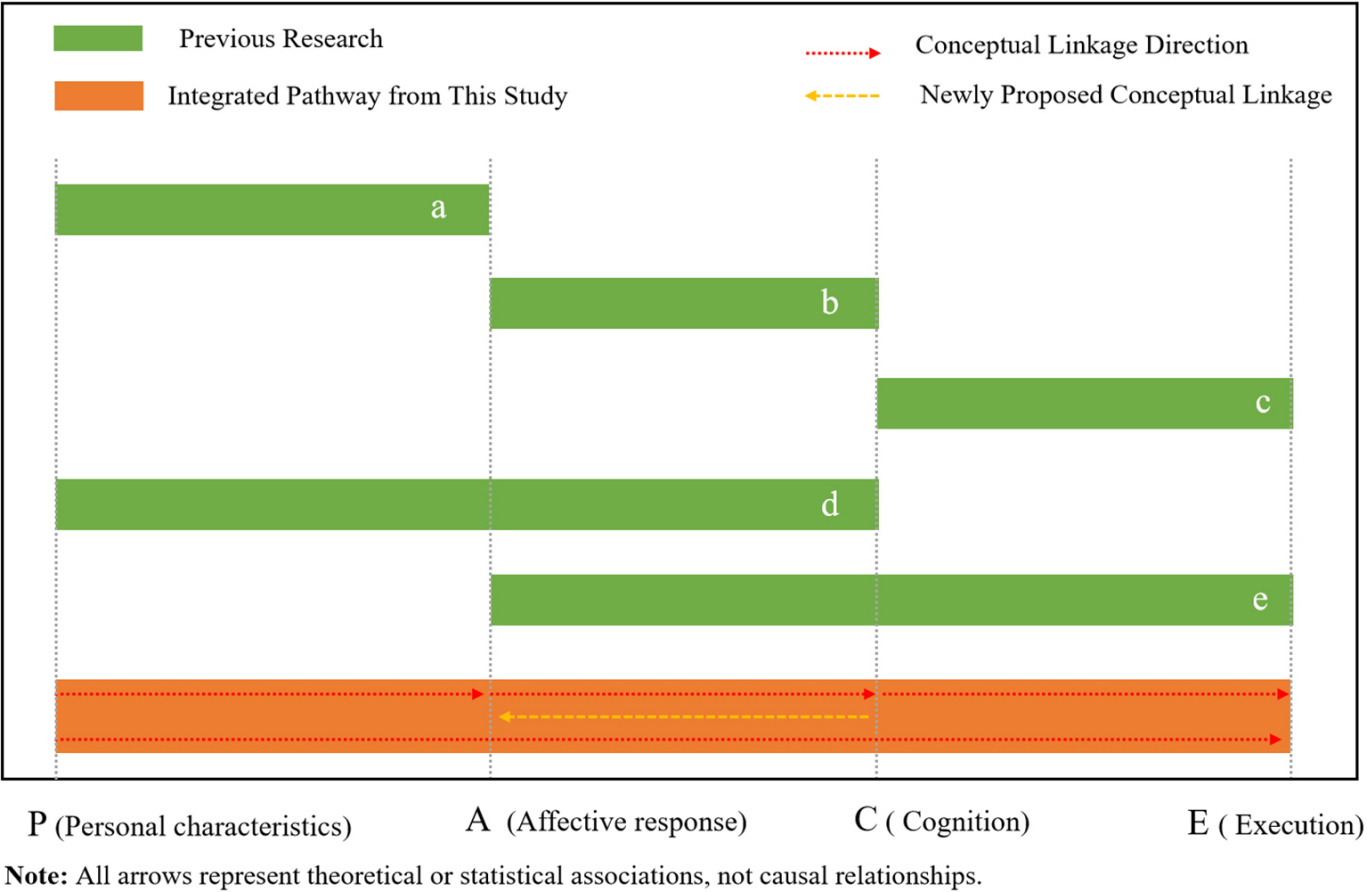
Conceptual integration of pathways within the I-PACE framework.

In summary, grounded in the I-PACE model, this study systematically examined the interrelations among psychological and behavioral pathways associated with DRP among middle-aged and older adults. The core contribution of this research lies not in confirming any single component in isolation, but in integrating previously fragmented relationships reported in earlier studies into a coherent framework. As illustrated in **Figure 4**, prior research has separately addressed the P→A (32), A→C (13), C→E (36), P→C (37), and the direct A→E association (38). However, these links have rarely been examined concurrently within a single empirical model. The present study identified and statistically supported all of these key pathways within an integrated framework and, notably, revealed a potential C→A reciprocal association, connecting these previously independent components into a conceptually dynamic P→A→C→E sequence. This integrated model offers a more unified perspective on how intrinsic psychosocial vulnerabilities may be associated with external digital risk awareness, providing a theoretically grounded and empirically supported framework for understanding the cognitive decision-making patterns of middle-aged and older adults in digital contexts.

### 5.2 Integrated pathways and overall mechanism

**Figure 5:**
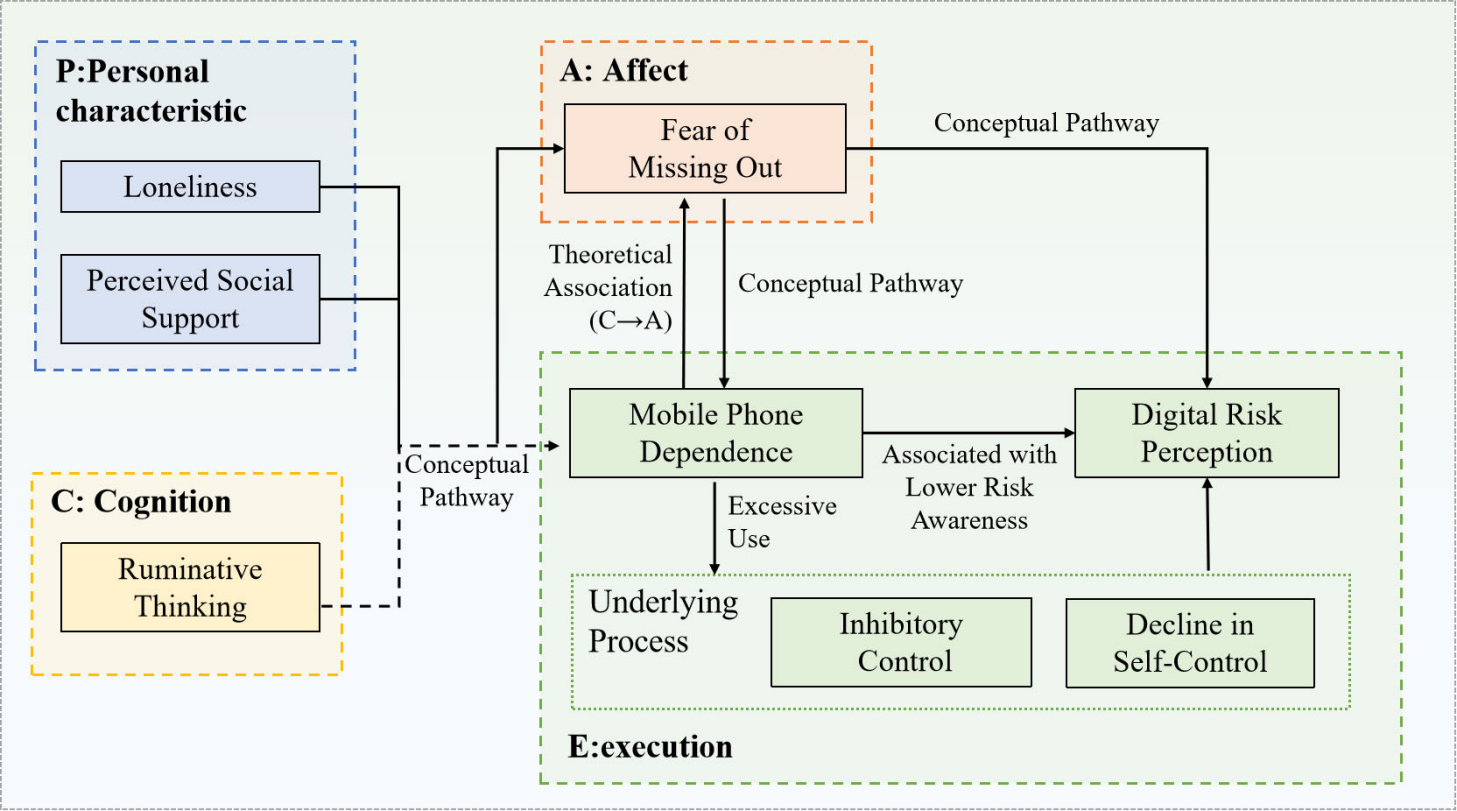
Conceptual model based on the I-PACE framework: digital risk perception. among middle-aged and older adults.

The pathways leading to digital risk perception, illustrated in **Figure 5**, reveal two core psychological routes identified through our empirical model. The first is the affective-driven pathway (P → A → E), in which traits such as loneliness are associated with lower digital risk perception through the emotional process of fear of missing out. The second, and more intricate, is the cognitive-mediated pathway (P → A → C → E), which emerged as the primary indirect association pattern in the present study.

As shown in **Figure 5**, the cognitive-mediated pathway outlines a coherent behavioral pattern whereby psychological needs-such as loneliness or low perceived social support-are statistically associated with emotional response of fear of missing out, which, in turn, relate to higher mobile phone dependence and lower digital risk perception. The model further suggests a potential C → A linkage, where mobile phone dependence appears to reinforce FoMO, representing a theoretically consistent but not empirically verified association.

The coexistence of these pathways, complemented by the theoretically plausible C → A linkage, delineates an integrative framework that conceptually connects psychosocial vulnerabilities to digital risk perception. By highlighting interrelated processes, the model advances a theoretical interpretation consistent with the process-oriented assumptions of the I-PACE framework.

### 5.3 Theoretical implications within the I-PACE framework

The results of this study can be interpreted within the I-PACE framework, which conceptualizes technology-related behaviors as outcomes of interactions among person, affective, cognitive, and executive components. The observed associations-such as the negative relationships between loneliness, fear of missing out, mobile phone dependence, and digital risk perception, and the positive association of perceived social support are consistent with this theoretical view. These findings indicate that emotional and behavioral tendencies are closely related to how individuals cognitively evaluate online risks.

By applying the I-PACE model to middle-aged and older adults, this study extends its contextual applicability to a population rarely examined in prior digital behavior research. Thereby refining its application beyond problematic smartphone and social media use to encompass digital risk perception and safety awareness. Rather than modifying the model, the present findings help clarify how its components manifest in later-life digital contexts, highlighting the relevance of psychosocial and affective processes in understanding digital risk awareness. In line with this interpretation, these findings suggest that theoretical frameworks should place greater emphasis on such age-specific psychosocial factors, rather than focusing solely on cognitive and executive components-a perspective consistent with broader research linking digital engagement to social well-being in later life. (39).

## 6 Conclusion

### 6.1 Major findings

Grounded in the I-PACE framework, this study identifies mobile phone dependence as a key behavioral correlate that is strongly associated with lower digital risk perception (DRP) among middle-aged and older adults in Chongqing, China. The findings further suggest that loneliness and low perceived social support may relate to reduced risk perception through affective (FoMO) and behavioral (mobile phone dependence) tendencies. Notably, SHAP analysis highlighted loneliness as the strongest contributing factor, followed by mobile phone dependence and FoMO, providing interpretive support for the structural equation modeling (SEM) results. Significant demographic differences were also observed, with urban residents reporting higher risk perception than rural counterparts, and mobile phone dependence increasing with age. Collectively, these results emphasize the importance of addressing psychological and behavioral risk factors, as well as bridging digital literacy disparities, in efforts to enhance online safety awareness among this population.

### 6.2 Limitations

First, the cross-sectional design precludes causal inference; the observed associations are correlational rather than directional, despite their theoretical alignment with the I-PACE framework. All reported “effects” in the SEM are interpreted as directional statistical paths rather than causal relationships.

Second, the sample consisted of Chinese adults aged 45 and above from Chongqing, representing a regional convenience sample with uneven age-group sizes-particularly fewer participants in the oldest group. These factors may limit the generalizability of the findings to broader populations.

Third, as all measures were self-reported, common-method bias cannot be fully ruled out, although a Harman’s single-factor test showed no single factor dominated the variance (33.6%). Residual bias may still exist; thus, future studies should combine self-reports with behavioral or experimental data to reduce potential bias.

Additionally, the relatively high ratio of estimated parameters to sample size (N = 356) may reduce the precision of parameter estimates. Future research should replicate the model using larger and more heterogeneous samples to ensure robustness.

Finally, the self-developed DRP scale showed very high internal consistency (α > 0.95), likely reflecting conceptual narrowness and sample homogeneity. Future studies should refine and validate the scale in broader populations to enhance its parsimony and generalizability.

### 6.3 Future research directions

Future research should prioritize longitudinal designs to verify the causal relationships proposed in this model and to explore the dynamic nature of feedback mechanisms over time. Expanding the sample to include participants from more diverse geographical and socioeconomic backgrounds would help enhance the external validity of the findings. Moreover, employing multi-method approaches, such as integrating smartphone usage logs with experimental tasks, could further enrich the data dimension.

In addition, although the SHAP analysis in this study provided valuable interpretive insights into the roles of psychological predictors, its application remains limited to a linear regression framework. Future research could further extend the implementation and model adaptation of SHAP, for example, by incorporating non-linear modeling approaches such as random forests and gradient boosting in future studies to better capture complex psychological mechanisms and variable interactions. Furthermore, refining SHAP visualization and interpretive strategies would help improve the clarity and transparency of the results, thereby promoting the broader application of this technique in psychological and behavioral research.

## Data Availability

The raw data supporting the conclusions of this article will be made available by the authors, without undue reservation.
