# Supplementary material for: Digital risk perception among middle-aged and older adults in China: a perspective from mobile phone dependence within the I-PACE model
Source: Front Psychiatry. 2026 Feb 6;17:1746285. doi: 10.3389/fpsyt.2026.1746285 (PMC12920230; doi:10.3389/fpsyt.2026.1746285)
Supplement: Supplementary file 1 [file DataSheet1.docx]

**Digital Risk Perception Questionnaire for Middle-aged and Older Adults**

Thank you for taking the time to complete this questionnaire. This survey is completely anonymous, and your responses will provide valuable insights for our research. Rest assured, your personal information will be kept strictly confidential and used solely for academic research purposes.

**Part 1: Demographic Information**

01. Your gender: A. Male; B. Female

02.Your age: A. 45-59 years; B. 60-69 years; C. 70 years and above

03.Education level: A. Primary school or below; B. Junior high school; C. High school or vocational school; D. College or above

04.Place of household registration: A. Urban; B. Rural

05.Your health status (self-assessed): A. Good; B. Average; C. Poor

**Part 2: Core Questions**

| **Dimension** | **Survey question** | **Question options** | | | | |
| --- | --- | --- | --- | --- | --- | --- |
|  |  | A | B | C | D | E |
| Knowledge | 1. I am aware that setting a lock screen password on my phone is an important way to protect personal privacy | Strongly Agree | Agree | Neutral | Disagree | Strongly Disagree |
|  | 1. I understand that “phishing websites” and “trojan viruses” may steal my personal information. | Strongly Agree | Agree | Neutral | Disagree | Strongly Disagree |
|  | 1. I believe that as long as I download apps from official app stores, my phone is very secure. | Strongly Disagree | Disagree | Neutral | Agree | Strongly Agree |
|  | 9.I understand that excessively sharing daily life details (such as travel plans and address) on social platforms like WeChat may pose risks. | Strongly Agree | Agree | Neutral | Disagree | Strongly Disagree |
| Attitude | 1. I believe that great caution is needed when connecting to free Wi-Fi in public places that doesn't require a password. | Strongly Agree | Agree | Neutral | Disagree | Strongly Disagree |
|  | 1. I am concerned that personal information such as photos and contacts on my phone may be secretly accessed by malicious apps. | Strongly Agree | Agree | Neutral | Disagree | Strongly Disagree |
|  | \| 1. I think that although cybersecurity issues exist, they mainly affect young people who are frequently online. \| \| --- \| | Strongly Disagree | Disagree | Neutral | Agree | Strongly Agree |
|  | 1. I believe that if I receive a suspicious “prize” or “refund” call or message, I should first doubt its authenticity. | Strongly Agree | Agree | Neutral | Disagree | Strongly Disagree |
| Practice | 1. I set different passwords for important accounts (such as WeChat and bank cards). | Strongly Agree | Agree | Neutral | Disagree | Strongly Disagree |
|  | 1. Before clicking on any unfamiliar links or scanning QR codes, I habitually verify whether the source is reliable. | Strongly Agree | Agree | Neutral | Disagree | Strongly Disagree |
|  | 1. I have scanned QR codes on event sites or flyers in order to receive small gifts or coupons from merchants. | Strongly Disagree | Disagree | Neutral | Agree | Strongly Agree |
|  | 1. I regularly check which apps on my phone have access to my location, photos, and other permissions, and I disable unnecessary authorizations. | Strongly Agree | Agree | Neutral | Disagree | Strongly Disagree |

**Thank you again for your participation. Wishing you a happy life!**
